# Identification of KRAP-expressing cells and the functional relevance of KRAP to the subcellular localization of IP_3_R in the stomach and kidney

**DOI:** 10.3892/ijmm.2012.1126

**Published:** 2012-09-17

**Authors:** TAKAHIRO FUJIMOTO, SENJI SHIRASAWA

**Affiliations:** 1 Department of Cell Biology, Faculty of Medicine and; 2 Central Research Institute for Advanced Molecular Medicine, Fukuoka University, 7-45-1 Nanakuma, Jonan-ku, Fukuoka 814-0180, Japan

**Keywords:** RAS-induced actin-interacting protein, inositol 1,4,5-trisphosphate receptor, immunohistochemical staining, protein-protein interaction, stomach, kidney

## Abstract

*KRAS*-induced actin-interacting protein (KRAP), originally identified as one of the deregulated genes expressed in colorectal cancer, participates under physiological conditions in the regulation of systemic energy homeostasis and of the exocrine system. We have recently found that KRAP is a molecule associated with inositol 1,4,5-trisphosphate receptor (IP_3_R) and is critical for the proper subcellular localization of IP_3_R in the liver and the pancreas. However, the expression of KRAP and its precise function in other tissues remain elusive. In this study, we aimed to identify the KRAP-expressing cells in mouse stomach and kidneys and to examine the relevance of KRAP expression in the regulation of IP_3_R localization in these tissues. In the stomach, double immunohistochemical staining for KRAP and IP_3_R demonstrated that KRAP was expressed along with the apical regions in the mucous cells and the chief cells, and IP_3_R3 was dominantly co-localized with KRAP in these cells. Furthermore, IP_3_R2 was also co-localized with IP_3_R3 in the chief cells. It is of note that the proper localization of IP_3_R3 and IP_3_R2 in the chief cells and of IP_3_R3 in the mucous cells were significantly abrogated in *KRAP*-deficient mice. In the kidneys, KRAP was expressed in both the apical and the basal regions of the proximal tubular cells. Intriguingly, *KRAP* deficiency abrogated the localization of IP_3_R1 in the proximal tubular cells. Finally, co-immunoprecipitation study in the stomachs and the kidneys validated the physical association of KRAP with IP_3_Rs. These findings demonstrate that KRAP physically associates with IP_3_Rs and regulates the proper localization of IP_3_Rs in the mucous cells and the chief cells of the stomach and in the proximal tubular cells of the kidneys.

## Introduction

Three inositol 1,4,5-trisphosphate receptor (IP_3_R) subtypes, IP_3_R1, IP_3_R2, and IP_3_R3, are differentially expressed among tissues (1–5) and function as the Ca^2+^ release channel on endoplasmic reticulum membranes (6–10). IP_3_R is regulated by many intracellular modulators, phosphorylation by kinases, and associated proteins (11–15).

*KRAS*-induced actin-interacting protein (KRAP) was originally identified as one of the deregulated expression gene in the colorectal cancer cell line, HCT116 (16). The previous studies using *KRAP*-knockout (*KRAP*-KO) mice demonstrate that KRAP participates in the regulation of systemic energy homeostasis (17) and of exocrine system (18). Among the adult mouse tissues, KRAP is ubiquitously expressed, with high levels in the pancreas, liver, and brown adipose tissues, and KRAP localizes in the restricted apical regions of the liver parenchymal cells and of the pancreatic exocrine acinar cells (19). Our recent findings indicate that KRAP associates with IP_3_R to regulate its proper subcellular localization in the mouse liver and the pancreas (20) as well as in immortalized cultured cell lines (21). Despite these advances, it remains largely unknown which cell types express KRAP among the other tissues including stomach and kidneys.

Herein, we performed immunohistological analysis and identified the exact KRAP-expressing cells in the stomach and the kidneys, and demonstrated that KRAP plays critical role in the regulation of the precise subcellular localization of IP_3_R in the mucous and the chief cells of the stomach and in the proximal tubular cells of the kidneys.

## Materials and methods

### Animals

All animals used in this study were treated in accordance with the guidelines of Fukuoka University. KRAP-knockout mice were generated as described previously (17).

### Immunohistochemical staining

Immunohistochemical staining was performed as described previously (19,20). Specific signals were detected by using rabbit polyclonal anti-KRAP antibody (19), mouse monoclonal anti-ZO-1 antibody (ZYMED), mouse monoclonal anti-IP_3_R3 antibody (610313) from BD Transduction Laboratories, rabbit polyclonal anti-IP_3_R2 antibody (AB3000) from Millipore, and rabbit polyclonal anti-IP_3_R1 antibody (ab5840) from Abcam.

### Immunoprecipitations and western blotting

Immunoprecipitations and western blotting were performed as described previously (19,20).

## Results

### Localization of KRAP protein in the adult mouse stomach

**Figure 1:**
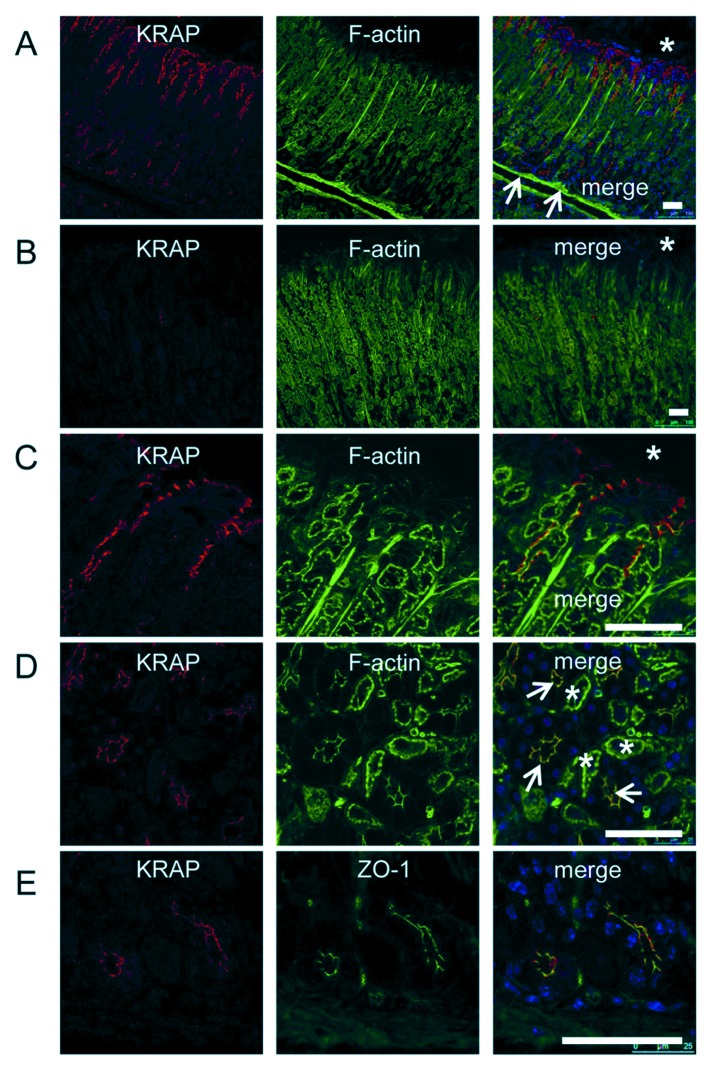
KRAP expression in the mucous cells and the chief cells of the mouse stomach. (A–D) Fluorescent confocal images of stomach sections for KRAP (red), filamentous actin (F-actin) with phalloidin (green), and the merged photo. Low magnification images from the pit region to the base region of gastric glands from wild-type (A) or *KRAP*-deficient (B) mice. Asterisk and arrows indicate gastric lumen and muscularis mucosae beneath the base region, respectively. (C) High magnification images of the pit region of gastric glands. Asterisk indicates gastric lumen. (D) High magnification images of the base regions of gastric glands. Asterisks and arrowheads indicate the parietal cells and the apical membranes of the chief cells, respectively. (E) Fluorescent confocal images of the base regions of gastric glands for KRAP (red), ZO-1 (green), and the merged photo. Blue, 4′,6-diamidino-2-phenylindole (DAPI) staining; scale bar, 50 *μ*m.

To examine the cellular distribution of KRAP protein in the adult mouse tissues, we performed immunohistochemical staining by using anti-KRAP antibody. In the stomach, strong KRAP immunoreactivity was restricted to the pit regions of gastric glands (Fig. 1A), whereas significant expression of KRAP was not detected in the muscularis mucosae beneath the gastric glands (Fig. 1A, arrows). The specificity of KRAP expression in the stomach was confirmed by using *KRAP*-KO tissue as a control (Fig. 1B). In the pit region of the gastric gland, where columnar surface mucous cells mainly exist (22), KRAP was localized beneath the apical membranes of the mucous cells (Fig. 1C). In the base region of the gastric glands, where zymogenic chief cells mainly exist, coronal plane of deeper gastric glands showed that KRAP was restricted to the apical regions of the chief cells (Fig. 1D, arrowheads), whereas KRAP was not detected in the parietal cells (Fig. 1D, asterisks). The distinction between the chief and the parietal cells was validated by ZO-1 staining as described (23), indicating that KRAP was expressed in the ZO-1-positive chief cells but not in the ZO-1-negative parietal cells (Fig. 1E).

### KRAP co-localized with IP_3_R in the stomach

**Figure 2:**
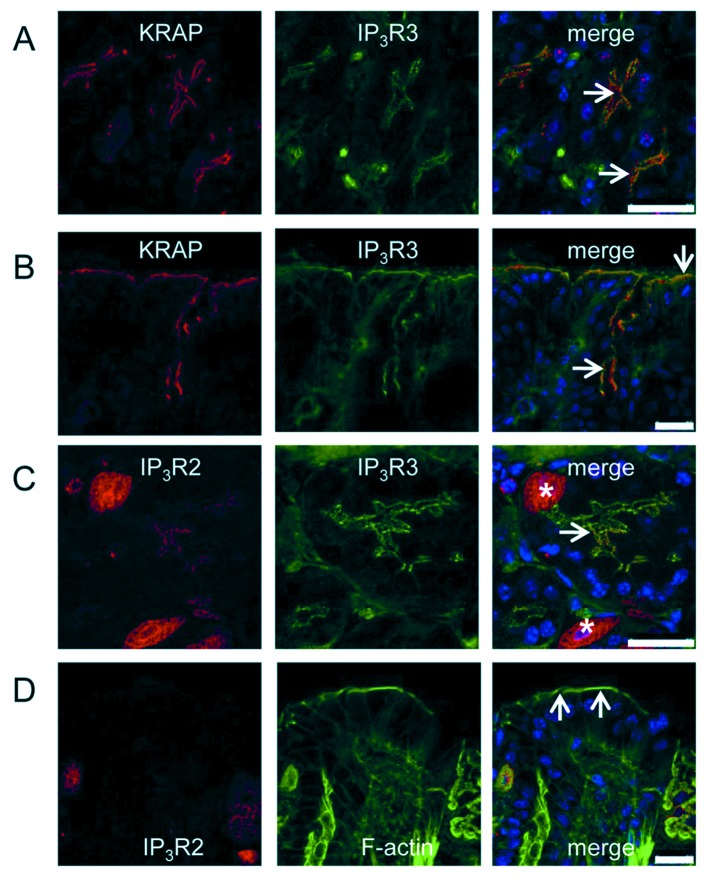
Colocalization of KRAP with IP_3_Rs in the chief cells and the mucous cells of the mouse stomach. (A) Fluorescent confocal images of the base region of gastric glands for KRAP (red), IP_3_R3 (green), and the merged photo. Arrows indicate the apical membranes of the chief cells. (B) Fluorescent confocal images of the pit region of gastric glands for KRAP (red), IP_3_R3 (green), and the merged photo. Arrows indicate the apical membranes of the mucous cells. (C) Fluorescent confocal images of the base region of gastric glands for IP_3_R2 (red), IP_3_R3 (green), and the merged photo. Asterisks and arrow indicate the parietal cells and the apical membranes of the chief cells, respectively. (D) Fluorescent confocal images of the pit region of gastric glands for IP_3_R2 (red), IP_3_R3 (green), and the merged photo. Arrows indicate the apical membranes of the mucous cells. Blue, 4′,6-diamidino-2-phenylindole (DAPI) staining; scale bar, 25 *μ*m.

Since we previously reported that KRAP associates with particular subtypes of IP_3_R in the liver and the pancreas (20), we examined whether KRAP in the stomach is also co-localized with IP_3_R. Double-immunostaining of the stomach for KRAP and IP_3_R3 revealed that KRAP was co-localized with IP_3_R3 in the apical regions of both the chief cells (Fig. 2A, arrows) and the mucous cells (Fig. 2B, arrows). Of note, IP_3_R2 co-existed with IP_3_R3 in the chief cells (Fig. 2C, arrow) but not in the parietal cells (Fig. 2C, asterisks). Furthermore, IP_3_R2 was not detected in the mucous cells (Fig. 2D, arrows). These results indicated that KRAP was co-localized with IP_3_R2 and IP_3_R3 in the chief cells and with IP_3_R3 in the mucous cells.

### Impaired localization of IP_3_R in the KRAP-deficient chief cells and the mucous cells

**Figure 3:**
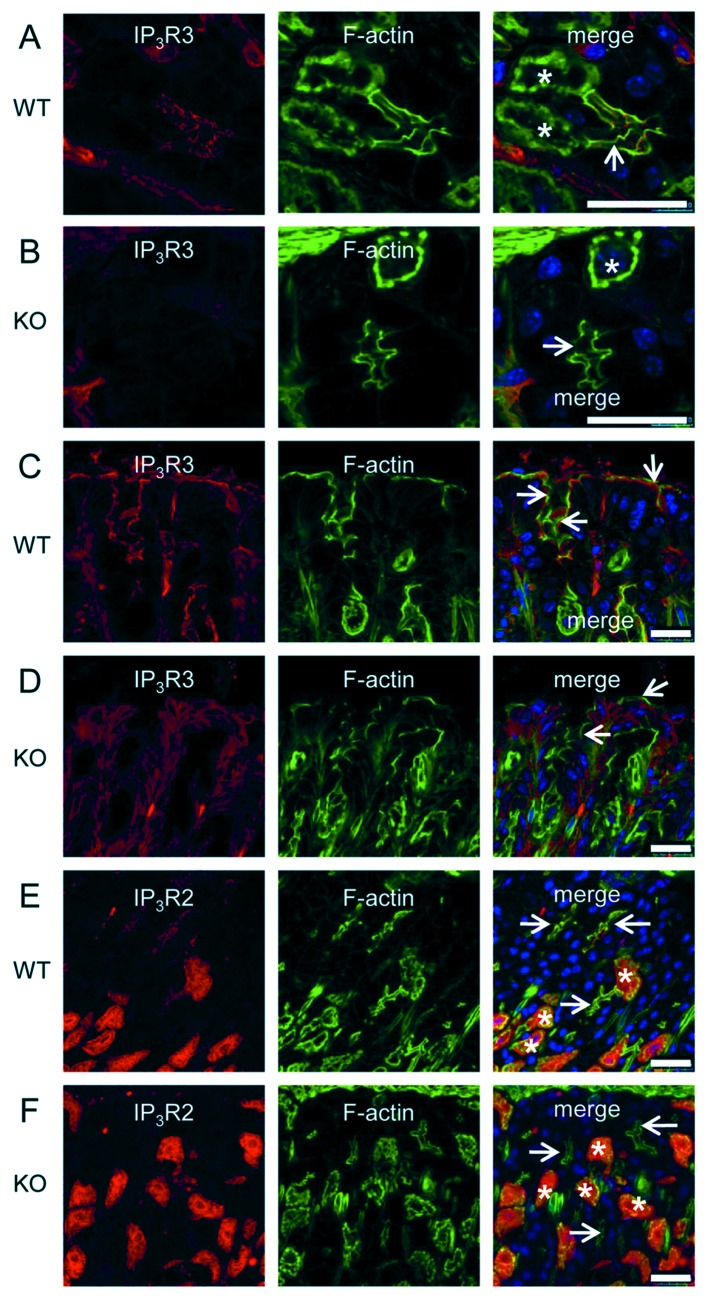
Impaired localization of IP_3_Rs in the *KRAP*-deficient chief cells and the mucous cells. (A and B) Fluorescent confocal images of the base region of gastric glands for IP_3_R3 (red), F-actin with phalloidin (green), and the merged photo from wild-type (WT) (A) or *KRAP*-deficient (KO) (B) mice. Asterisks and arrows indicate the parietal cells and the apical membranes of the chief cells, respectively. (C and D) Fluorescent confocal images of the pit region of gastric glands for IP_3_R3 (red), F-actin (green), and the merged photo from WT (C) or KO (D) mice. Arrows indicate the apical membranes of the mucous cells. (E and F) Fluorescent confocal images of the base region of gastric glands for IP_3_R2 (red), F-actin (green), and the merged photo from WT (E) or KO (F) mice. Asterisks and arrows indicate the parietal cells and the apical membranes of the chief cells, respectively. Blue, 4′,6-diamidino-2-phenylindole (DAPI) staining; scale bar, 25 *μ*m.

We addressed the functional relevance of KRAP to the proper localization of IP_3_R by using *KRAP*-KO mice. IP_3_R3 was located in the apical region of the chief cells (Fig. 3A, arrow) and of the mucous cells (Fig. 3C, arrows) in the wild-type (WT) mouse stomach, whereas the restricted localization of IP_3_R3 appeared to be diminished in the *KRAP*-KO stomach (Fig. 3B, arrow; 3D, arrows). Furthermore, IP_3_R2 was detected in both the chief cells (Fig. 3E, arrows) and the parietal cells (Fig. 3E, asterisks) in the WT stomach, whereas the localization of IP_3_R2 in the *KRAP*-KO stomach was impaired in the chief cells (Fig. 3F, arrows) but not in the parietal cells (Fig. 3F, asterisks). Thus, KRAP plays critical role in the regulation of the proper localization of IP_3_R2 and IP_3_R3 in the chief cells and of IP_3_R3 in the mucous cells.

### KRAP expression and its contribution to the localization of IP_3_R1 in the proximal tubules of the mouse kidney

**Figure 4:**
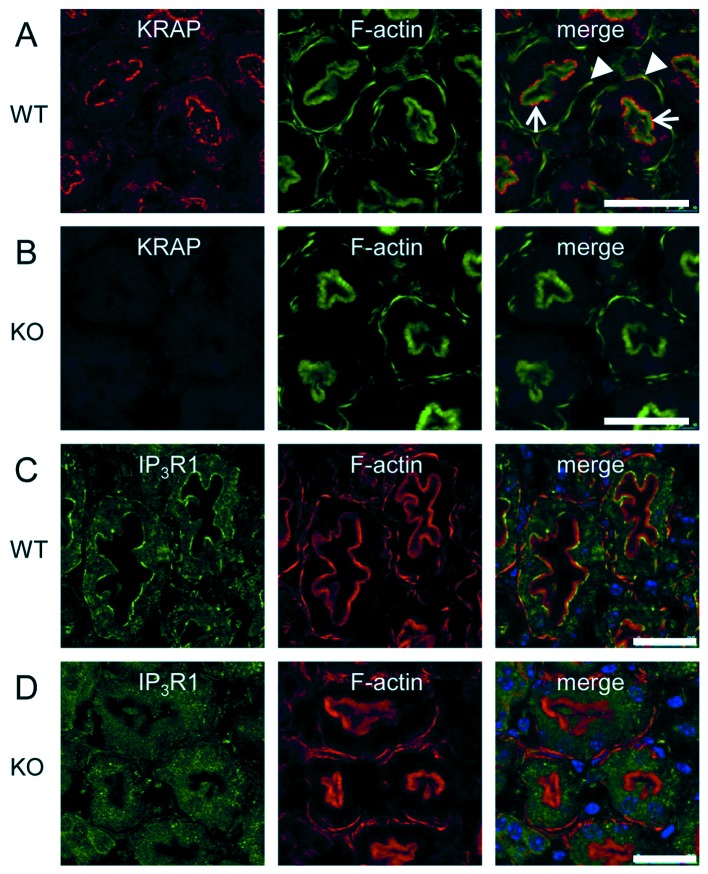
KRAP expression and its contribution to the localization of IP_3_R1 in the proximal tubules of the mouse kidney. (A and B) Fluorescent confocal images of the proximal tubules of kidney for KRAP (red), F-actin (green), and the merged photo from wild-type (WT) (A) or *KRAP*-deficient (KO) (B) mice. Arrowheads and arrows indicate the basolateral and the apical regions of the proximal tubules, respectively. (C and D) Fluorescent confocal images of the proximal tubules of kidney for IP_3_R1 (green), F-actin (red), and the merged photo from WT (C) or KO (D) mice. Blue, 4′,6-diamidino-2-phenylindole (DAPI) staining; scale bar, 25 *μ*m.

To examine the cellular distribution of KRAP protein in the adult mouse kidneys, we performed immunohistochemical staining by using anti-KRAP antibody. The specificities of the signals were validated by comparing the immunoreactivities of WT and *KRAP*-KO mouse tissues. In the WT kidneys, intense immunoreactivities were observed in the renal proximal tubules (Fig. 4A) but not in the renal distal tubules (data not shown). On the other hand, significant immunoreactive signal was not detected in the proximal tubules in the *KRAP*-KO mice (Fig. 4B). Taken together, these results indicate that KRAP was exactly expressed in the proximal tubules. The proximal tubules were identified by the presence of the brush-border stained with phalloidin (Fig. 4A and B). Immunostaining in the proximal region showed that KRAP was accumulated beneath the brush-border (Fig. 4A, arrows) and KRAP was also detected in the basolateral actin bundles (Fig. 4A, arrowheads). We next examined which subtypes of IP_3_R, IP_3_R1, IP_3_R2, and IP_3_R3, expressed in the proximal tubular cells, revealing that IP_3_R1 (Fig. 4C) but not IP_3_R 2 or IP_3_R3 (data not shown) was detected in the beneath the brush-border and in the basolateral actin bundles. Finally, we addressed the functional relevance of KRAP expression in the proximal tubular cells to the regulation of IP_3_R localization. It is of note that the restricted localization of IP_3_R1 detected in the WT mouse kidney (Fig. 4C) was disturbed in the *KRAP*-KO mouse kidney (Fig. 4D). Thus, KRAP plays critical role in the regulation of the proper localization of IP_3_R1 in the proximal tubular cells.

### KRAP interacts with IP_3_R1 in the kidneys and with IP_3_R3 in the stomach

**Figure 5:**
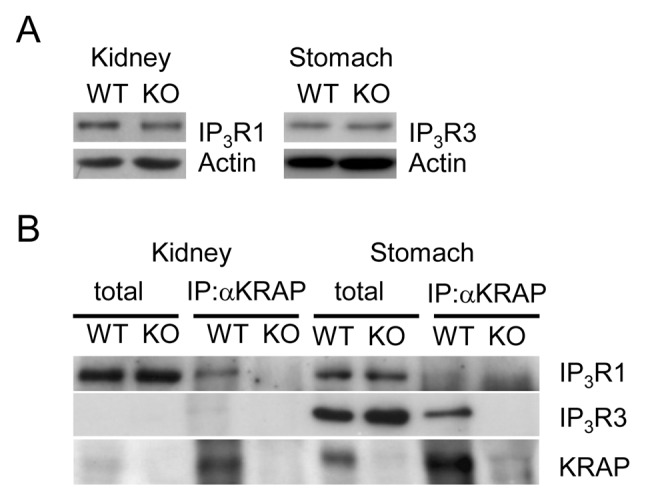
KRAP interacts with IP_3_R1 in the kidney and with IP_3_R3 in the stomach. (A) Western blots showing comparable expression levels of IP_3_R1 in the kidney (left) or of IP_3_R3 in the stomach (right) between *KRAP*-deficient (KO) and wild-type (WT) mice. (B) Anti-KRAP (αKRAP) immunoprecipitations were performed using mouse kidneys and stomachs from WT or KO mice, followed by western blotting with anti-KRAP, anti-IP_3_R1, or anti-IP_3_R3 antibodies. total, total lysate;IP, immunoprecipitation; α, anti-.

As described above, immunohistochemical signals for particular IP_3_R subtypes in the *KRAP*-KO mouse kidneys or the stomach were abrogated, leading us to check the expression levels of IP_3_R between the WT and *KRAP*-KO mouse tissues. Normal expression levels of IP_3_R1 and IP_3_R3 were detected in the *KRAP*-KO mouse kidney and the stomach, respectively, compared with the WT mouse tissues (Fig. 5A), suggesting that mislocalizations but not deregulated expressions of IP_3_R occur in the *KRAP*-KO mouse kidneys and the stomach. Next, to examine the physical association of KRAP with IP_3_R, we performed co-immunoprecipitations by anti-KRAP antibody in the kidneys or the stomach, in which we could not evaluate the specific association of IP_3_R2 with KRAP due to lack of IP_3_R2-specific antibody available for western blotting. In the preparations from the WT mouse tissues, KRAP precipitates IP_3_R1 and IP_3_R3 in the kidney and the stomach, respectively (Fig. 5B). The specificity of co-immunoprecipitations of IP_3_R was confirmed by using *KRAP*-KO mouse tissue as a control (Fig. 5B). Thus, KRAP physically interacts with IP_3_R1 in the kidneys and with IP_3_R3 in the stomach.

## Discussion

In this study, we demonstrated that KRAP protein expression and the subcellular localization was restricted beneath the apical and/or basolateral membranes in specific cell types of the stomach and the kidneys, in which KRAP physically associated with particular IP_3_R subtype(s). In the *KRAP*-KO mouse stomach and the kidneys, the polarized localization of IP_3_R was impaired, indicating that KRAP plays critical roles in the regulation of the proper subcellular localization of IP_3_R in the stomach and the kidneys.

Notably, KRAP as well as IP_3_R3 proteins were polarized beneath the apical membranes facing the gastric gland lumen and were absent in the parietal cells (Fig. 1), suggesting an association of these proteins with chief cell functions including pepsinogen secretion (22–24). From this view point, KRAP expression and the localization beneath the apical membranes of the pancreatic acinar cells (19), another type of zymogen cells, may suggest a similar role for KRAP in the stomach and the pancreas. Considering the fact that KRAP physically interacts with IP_3_R to regulate its proper localization in these tissues, stomach (Figs. 2, 3 and 5) and pancreas (20), and that double-knockout of IP_3_R2 and IP_3_R3 in mice revealed a failure in secretory function in the pancreas (25), KRAP seems to be involved in the exocrine systems. Actually, the pancreatic acinar cells in *KRAP*-KO mice showed an increased amount of zymogen granules, although they seemed to maintain the proper physiological agonist-induced exocytosis (18). Thus, exact functional relevance of KRAP and its interaction with IP_3_R to the exocrine systems in the pancreas and the stomach should await future studies.

It is of note that KRAP was restricted to both the apical region and the basolateral region of the proximal tubular cells of the kidneys (Fig. 4), and that KRAP physically associated with IP_3_R1 in the kidneys (Fig. 5). Furthermore, our previous study showed that KRAP was distributed along the bile canaliculi of hepatocytes and underneath the apical membrane of pancreatic acinar cells (19). All these KRAP localizations in the distinct tissues examined are restricted to epithelial cell types bearing well-developed cell polarity, cell-cell junction and microvilli, where transports of various substances between epithelial cells and extracellular spaces, exocrine space or blood stream occur (22,26–28). Since *KRAP*-KO mice displayed profound metabolic disorders after birth without developmental defects, and certain systemic inter-tissue dysregulations appeared to underlie the metabolic phenotypes (17), KRAP might play physiological roles in secretion and/or absorption functions after birth rather than in developmental events.

Renal proximal tubules serve the reabsorption of the bulk of substances filtered in the glomeruli and the excretion (26,29). These two opposite transports are accomplished by the coordinated action of ion channels and transporters located in the brush border membrane and basolateral membrane (29–31). Thus, the polarized expression of these membrane proteins is crucial for the function of the proximal tubules. Based on the findings that KRAP protein possesses characteristic features like scaffolding protein, such as polarized localization and transporting of IP_3_R, potential functional relevance of KRAP to these processes would be suspected.

In conclusion, we identified the exact KRAP-expressing cells in the stomach and the kidneys, and found that KRAP physically associates with IP_3_R to regulate its proper subcellular localization *in vivo*. Considering the KRAP function as an IP_3_R regulator and the importance of KRAP in energy homeostasis *in vivo*, further research on the exact relevance of the association between KRAP and IP_3_R to the biological phenomena will lead to a better understanding of physiological metabolic processes.
